# Critical Role of Light in the Growth and Activity of the Marine N_2_-Fixing UCYN-A Symbiosis

**DOI:** 10.3389/fmicb.2021.666739

**Published:** 2021-05-05

**Authors:** Marine Landa, Kendra A. Turk-Kubo, Francisco M. Cornejo-Castillo, Britt A. Henke, Jonathan P. Zehr

**Affiliations:** Ocean Sciences Department, University of California, Santa Cruz, Santa Cruz, CA, United States

**Keywords:** UCYN-A, N_2_-fixing symbiosis, *nifH*, diel cycle, host photosynthesis, circadian rhythm, light dark cycles, gene expression

## Abstract

The unicellular N_2_-fixing cyanobacteria UCYN-A live in symbiosis with haptophytes in the *Braarudosphaera bigelowii* lineage. Maintaining N_2_-fixing symbioses between two unicellular partners requires tight coordination of multiple biological processes including cell growth and division and, in the case of the UCYN-A symbiosis, N_2_ fixation of the symbiont and photosynthesis of the host. In this system, it is thought that the host photosynthesis supports the high energetic cost of N_2_ fixation, and both processes occur during the light period. However, information on this coordination is very limited and difficult to obtain because the UCYN-A symbiosis has yet to be available in culture. Natural populations containing the UCYN-A2 symbiosis were manipulated to explore the effects of alterations of regular light and dark periods and inhibition of host photosynthesis on N_2_ fixation (single cell N_2_ fixation rates), *nifH* gene transcription, and UCYN-A2 cell division (fluorescent *in situ* hybridization and *nifH* gene abundances). The results showed that the light period is critical for maintenance of regular patterns of gene expression, N_2_ fixation and symbiont replication and cell division. This study suggests a crucial role for the host as a producer of fixed carbon, rather than light itself, in the regulation and implementation of these cellular processes in UCYN-A.

## Introduction

Marine phytoplankton are comprized of diverse eukaryotic algae and cyanobacteria and are responsible for approximately half of the fixation of atmospheric CO_2_ into organic matter on Earth (Field et al., 1998). This primary productivity is dependent on the biogeochemical cycling of nutrients. In particular, biologically available nitrogen (N) is often in short supply despite the vast reservoir of atmospheric dinitrogen gas (N_2_). Biological N_2_ fixation, i.e., the conversion of N_2_ to biologically available ammonium, is a significant source of new N in the surface ocean that supports primary production (Karl et al., 1997; Shiozaki et al., 2013). It is performed by a limited, albeit diverse, number of Archaea and Bacteria taxa called diazotrophs. Among them, the unicellular cyanobacterium UCYN-A group contributes significantly to oceanic N_2_ fixation (Church et al., 2005a; Martnez-Prez et al., 2016). This group encompasses closely related sublineages that are symbiotic with several different species of single celled haptophytes in the *Braarudosphaera bigelowii* clade (Thompson et al., 2012, 2014; Hagino et al., 2013). Since its discovery, the UCYN-A symbiosis has been found in a wide variety of oceanic habitats (Farnelid et al., 2016) including areas that were previously not considered important for diazotrophy, such as N-rich coastal ecosystems (Mulholland et al., 2012; Tang and Cassar, 2019) and the Arctic Ocean (Harding et al., 2018; Shiozaki et al., 2018).

UCYN-A transfers fixed N to the host in exchange for fixed carbon (C), which was demonstrated with isotope experiments using nanoscale secondary ion mass spectrometry (nanoSIMS) analyses (Thompson et al., 2012; Krupke et al., 2013; Mills et al., 2020). The UCYN-A genome unveiled an extremely streamlined metabolism, missing many important pathways and enzymes that are typical for cyanobacteria and necessary for a free-living lifestyle. UCYN-A lacks the oxygen-evolving photosystem II (PSII), Rubisco and Calvin cycle enzymes, the entire tricarboxylic acid (TCA) cycle and various enzymes for branched chain amino acid and nucleotide synthesis (Zehr et al., 2008; Tripp et al., 2010; Bombar et al., 2014). Thus, it is likely that the host provides several essential metabolites to the symbiont and that the symbiosis is obligatory, at least for the symbiont (Zehr et al., 2016).

In eukaryotic phytoplankton and cyanobacteria, metabolism and growth are coordinated with the light and dark periods of the daily cycle, which is reflected by oscillations in gene expression (Welkie et al., 2019; Becker et al., 2021). In N_2_-fixing cyanobacteria, such metabolic synchronization is particularly important for light-driven photosynthesis and supply of energy for N_2_ fixation. N_2_ fixation is inhibited by oxygen and diazotrophic cyanobacteria typically separate N_2_ fixation from oxygen-evolving photosynthesis either temporally or spatially in specialized cells called heterocysts (Fay, 1992; Thompson and Zehr, 2013). Most unicellular diazotrophic cyanobacteria, including marine planktonic species, are nighttime N_2_-fixers (Zehr et al., 2001; Berman-Frank et al., 2003; Tuit et al., 2004; Mohr et al., 2010b; Wilson et al., 2017). In these species, light enables the production of carbohydrates that are later used as energy sources to fuel nighttime N_2_ fixation (Dron et al., 2012). UCYN-A is unusual among unicellular N_2_-fixing cyanobacteria because it fixes N_2_ during the day (Krupke et al., 2013; Zehr et al., 2016). In fact, the whole genome expression pattern in UCYN-A, including genes for N_2_ fixation, is more similar to that of daytime N_2_-fixers than of the more closely related unicellular cyanobacteria (Muoz-Marn et al., 2019). It is not known what controls the unusual N_2_ fixation timing in UCYN-A. Although it lacks most proteins involved in photosynthesis, UCYN-A retains and expresses photosystem I (PSI) genes in a diel pattern, and depends on its host for photosynthate, which suggests direct and indirect roles for light in N_2_ fixation and UCYN-As metabolism in general. Besides photosynthesis and N_2_ fixation, the UCYN-A symbiosis faces several physiological challenges with respect to cell growth and division. The host and UCYN-A cells must coordinate growth and cell division to avoid outgrowing one another and losing their partnership. UCYN-A has been shown to express cell division genes at the end of the light period and divide a few hours later, prior to the host cell division (Cabello et al., 2016; Muoz-Marn et al., 2019), but the mechanisms that control this process are unknown.

In this study, we manipulated coastal microbial communities containing the UCYN-A2 symbiosis to assess the influence of the daily light-dark cycle on the symbiosis growth and metabolism. First, we manipulated light conditions to investigate links between light and daytime N_2_ fixation by UCYN-A2. Second, we used a photosynthesis inhibitor specific to PSII to examine the response of the symbiont to arrested host C fixation. We sampled at regular time intervals (every 4 h) over a 52-h experimental time course, and combined targeted *nifH* gene and transcript quantification, microscopic observations, and cell-specific C and N_2_ fixation rate measurements to determine the responses of the UCYN-A2 symbiosis to altered light-dark cycles and host photosynthesis at high temporal resolution.

## Materials and Methods

### Experimental Setup

**FIGURE 1:**
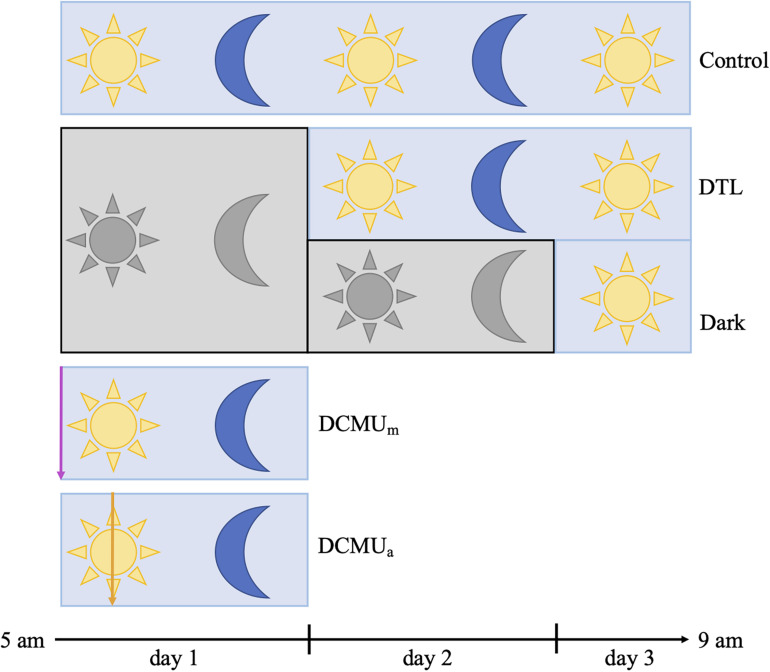
Schematic representation of experimental conditions. Blue rectangles represent clear incubators and gray rectangles represent opaque incubators. Yellow sun and blue moon represent normal light-dark conditions achieved with clear incubators, and gray sun and moon represent complete darkness achieved with opaque incubators. In the two conditions involving a dark phase, bottles were placed in opaque incubators at the beginning of the experiment and then moved to clear incubators at 5 a.m. on day 2 (Dark then light, DTL) or on day 3 (Dark). For incubations receiving DCMU, the purple arrow represents DCMU addition before 5 a.m. (DCMU_*m*_) and the orange arrow represents DCMU addition at 1 p.m. (DCMU_*a*_). See section Materials and Methods for additional description of incubation and sampling procedures.

Incubations of natural marine microbial communities were set up on the Ellen Browning Scripps Memorial Pier at the Scripps Institution of Oceanography (SIO) in La Jolla, CA, United States in May 2018. Surface water was sampled off the pier using a pneumatic pump, passed through 150-m nitex mesh (Wildco, Yulee, FL, United States) and collected into a 40 L polycarbonate (PC) carboy, then gently distributed into 1 and 2 L clear PC bottles. All bottles and tubing were washed with 10% HCl and rinsed three times with milli-Q water, then rinsed three times with seawater immediately before use. Water collection and bottle filling took place between 10 p.m. and 4 a.m. Bottles were placed in clear or opaque incubators (Figure 1 and Supplementary Figure 1) equipped with HOBO data loggers (Onset, Bourne, MA, United States) to monitor light and temperature, and flushed continuously with surface seawater to keep the bottles at *in situ* temperature. Ambient temperature dropped from an average 19.8 4.2C during the day to 15.6 0.6C during the night over the course of the 52-h experiment. In the incubators, average nighttime temperatures (16.1 1.1 and 16.3 1.2C in clear and opaque incubators, respectively) were only slightly lower than during the day, indicating that temperature in the experimental system was successfully maintained as close as possible to *in situ* levels (Supplementary Figure 2). Clear containers were fitted with neutral density screening to attenuate incident light to ca. 20% of the surface irradiance, while the opaque containers were closed with an opaque lid to prevent exposure to light. Additionally, bottles in the opaque containers were placed in pairs in dark plastic bags to minimize exposure during sampling. The opaque incubators remained dark, except during brief opening for sampling (Supplementary Figure 3). Light levels in the clear incubators reached an average of 13 10% of incident light during daytime (Supplementary Figure 3).

### Experimental Timeline and Conditions

Five experimental conditions, Control, Dark, DTL (dark then light), DCMU_*m*_, and DCMU_*a*_ were implemented as illustrated in Figure 1 and Supplementary Figure 1. At 5 a.m. on day 1, fourteen 2 L bottles and twelve 1 L bottles were spiked with a solution of 3-(3,4-dichlorophenyl)-1,1-dimethylurea (DCMU, Sigma-Aldrich, St. Louis, MO, United States) at a final concentration of 100 g L^1^ and placed in clear incubators (DCMU_*m*_). The process was repeated at 1 p.m. on day 1 with twelve 2 L bottles and ten 1 L bottles in the clear incubators (DCMU_*a*_).

After the first 24 h of incubation, at 5 a.m. on day 2, sixteen 2 L bottles and fourteen 1 L bottles were moved from opaque incubators to clear incubators (Dark then Light). After the first 48 h of incubation, at 5 a.m. on day 3, the two bottles remaining in opaque incubators were moved to a clear incubator (Dark). In total, four different conditions were run in parallel during day 1 (Control, Dark, DCMU_*m*_, and DCMU_*a*_) while three different conditions were run during day 2 (Control, DTL, and Dark, Figure 1).

At 5 a.m. on days 1 and 2, duplicate 2 and 1 L bottles from each condition were spiked with ^15^N_2_-enriched seawater and NaH^13^CO_3_-enriched seawater and incubated for 24 h (Supplementary Figure 1). ^15^N_2_-enriched seawater (Mohr et al., 2010a) was prepared based on protocols described in Klawonn et al., 2015 (see also Supplementary Material). Stock NaH^13^CO_3_-enriched seawater (353 mM) was prepared by dissolving 6 g NaH^13^CO_3_ (Cambridge Isotope Laboratories) into 200 mL of sterile filtered site water. Experimental treatments were spiked with 10% (v/v) ^15^N_2_-enriched filtered seawater and 224 L NaH^13^CO_3_-enriched seawater (35 M final concentration) at the beginning of each 24-h incubation period. This was equivalent to a ca. 2% ^13^C enrichment. To verify the ^15^N atom% enrichment, the ^15^N_2_-enriched seawater was immediately subsampled (prior to spiking) into duplicate 12 ml Exetainers (Labco, Lampeter, Ceredigion, United Kingdom), which were stored at room temperature. Nitrogen isotopes (i.e., N masses equivalent to 28, 29, and 30) were measured via membrane inlet mass spectrometer (MIMS) analysis at the University of Hawaii according to Wilson et al. (2012). Calibration of the MIMS was achieved by the analysis of a 1 L reservoir of air-equilibrated filtered (0.2 m) seawater with a known salinity and a temperature of 23C (Bttjer et al., 2017). The final ^15^N atom % enrichment in the seawater incubations averaged 3.9 0.2 (day 1) and 5.7 0.1 (day 2).

### DNA/RNA and CARD-FISH Sampling

Duplicate bottles were retrieved at the onset of the experiment at 5 a.m. on day 1, after which duplicate bottles from all conditions were retrieved every 4 h until 9 a.m. on day 3 (Supplementary Figure 1). For DNA and RNA analysis, 1 L from duplicate 2 L bottles from each experimental condition was gently passed through a Sterivex unit (Millipore, Burlington, MA, United States) using a peristaltic pump. Filtration time never exceeded 15 min. Sterivex units were capped, flash frozen and stored in liquid nitrogen in the field then stored at 80C until further processing. For catalyzed reporter deposition-fluorescence in situ hybridization (CARD-FISH) analysis, 95 mL subsamples were removed from duplicate 1 L bottles from each experimental condition. At 5 a.m. on days 2 and 3, those samples were taken from the 1 L incubations enriched with ^15^N/^13^C 24 h prior to sampling. CARD-FISH samples were fixed with 5 mL 37% formaldehyde (1.85% final concentration) and placed in the dark at 4C for at least 1 h. Fixed samples were gently filtered onto a PC membrane (25 mm diameter, 0.6 m pore size, Millipore) using a vacuum pump. Filters were stored at 80C until further processing.

### Bulk N_2_ and C Fixation Rate Sampling and Determination

In parallel with samples for cell-specific rate analysis, 400 mL samples for particulate organic C (POC) and N (PON) were filtered through precombusted (4 h at 450C) 25 mm Whatman GF/F filters (Millipore) and stored at 20C. Filters were then dried at 60C, fumed with concentrated HCl, then dried at 60C for 24 h, before being packed into tin capsules (Costech Analytical Technologies Inc., Valencia, CA, United States). Samples for the analysis of ^15^N and ^13^C enrichment of the particulate organic matter were processed on an Elemental Combustion System (Costech Analytical Technologies) interfaced to a Thermo Finnigan Delta V Advantage isotope ratio mass spectrometer (Thermo Fisher Scientific, Waltham, MA, United States) at the SOEST Biogeochemical Stable Isotope Facility at the University of Hawaii, Manoa. The limit of detection (LOD), estimates of error, and minimum quantifiable rates (MQR) were calculated as in Gradoville et al. (2017) and are shown in Supplementary Table 1.

### RNA/DNA Extraction

Nucleic acids were extracted using a cell lysis protocol followed by the AllPrep DNA/RNA Mini kit (Qiagen, Hilden, Germany). Frozen filters were quickly transferred to bead beating tubes containing a mixture of 0.1- and 0.5-mm glass beads (Biospec Products, Inc., Bartlesville, OK, United States) and 1 mL of pre-warmed RLT+ lysis buffer (Qiagen) containing -mercaptoethanol (1% final concentration) and RNA standards (ERCC ExFold RNA Spike-In, final concentration 0.04X, Invitrogen^TM^, Thermo Fisher Scientific). Cell lysis was performed using three freeze-thaw cycles and two 2-min bead beating cycles (Mini-Beadbeater-96; Biospec Products, Inc.). Extraction was then performed following the manufacturers protocol, including on-column DNAse treatment (Qiagen) of the RNA fraction. The purity of the isolated DNA and RNA was checked using a Nanodrop (Thermo Fisher Scientific) and concentrations were determined using a Qubit (Invitrogen^TM^, Thermo Fisher Scientific). The quality and integrity of the RNA were assessed using a Bioanalyzer (Agilent, Santa Clara, CA, United States). A fraction of the RNA was immediately converted into complementary DNA (cDNA) using the High Capacity cDNA Reverse Transcription Kit (Applied Biosystems^TM^, Thermo Fisher Scientific). DNA and cDNA samples were stored at 20C while RNA samples were stored at 80C until further processing.

### UCYN-A2 *nifH* Gene Copy and Transcript Quantification (ddPCR)

Patterns in *nifH* gene and transcript abundances were investigated using digital droplet PCR (ddPCR), which enables standard-independent absolute quantification. The PCR reaction was prepared by adding DNA or cDNA templates to the ddPCR Supermix (Bio-Rad, Hercules, CA, United States) mixed with forward and reverse primers (0.4 M final concentration) and a probe (0.2 M final concentration), all targeting the UCYN-A2 *nifH* gene (Thompson et al., 2014). All samples were run in triplicates in 96-well microplates along with at least three no-template controls. Additional reactions were also run for the RNA used to generate cDNA which ruled out potential genomic DNA contamination. Plates were sealed, vortexed and centrifuged briefly, and droplets were generated using an Auto Droplet Generator (Bio-Rad) following the manufacturer instructions. The PCR reaction was set up as follows: 10 min at 95C, then 40 cycles of 30 s at 94C and 1 min at 64C, and a final step of 10 min at 98C. Positive droplet counts were performed using the Automated Droplet Reader (Bio-Rad), and data were processed with the QuantaSoft Analysis Pro software (Bio-Rad). Only runs with no positive droplet counts in the no-template controls were further processed, and sample wells in which the total number of droplets generated was below 10,000 were dropped. Positive droplet counts in each sample were then converted into UCYN-A2 *nifH* gene copies L^1^ or transcripts L^1^. After transcript normalization by gene abundance, periodic expression patterns were tested using the RAIN package in R (Thaben and Westermark, 2014).

### UCYN-A2 Symbiosis Cell-Specific C and N_2_ Fixation Rate Determination

A subset of fixed, frozen samples were processed for CARD-FISH as described in Cabello et al. (2016) with small modifications (see Supplementary Material). Processed filter sections mounted on slides were kept at 20C until microscopy observation. Individual symbioses were observed with an epifluorescence microscope (Zeiss Axioplan) using filters to detect the CY-3 signal corresponding to the symbiont, the Alexa-488 signal corresponding to the host, and to visualize DNA through DAPI signal.

Selected samples were transferred to silicon wafer grids for analysis of C and N isotopic composition of individual UCYN-A2 host associations using nanoSIMS according to Dekas and Orphan (2011). The location of at least 10 UCYN-A2-host associations per sample were mapped for analysis on a Cameca NanoSIMS 50 L at the Stanford Nano Shared Facilities. Wafers were loaded in the instrument and mapped symbioses were localized. Each symbiosis was processed individually, and instrument tuning was checked and adjusted between each cell. A 2-min exposure to a cesium ion (Cs^+^) beam was applied to each cell before analysis to remove contaminants. Sample surfaces were rastered with a 16 keV Cs^+^ beam and a current between 2 and 4 pA over a 15 15 m area. Data were simultaneously collected for ^12^C, ^13^C, ^12^C^14^N, and ^12^C^15^N secondary anions along with a secondary electron image, in 30 planes per cell. Raw data processing was performed using look@nanosims (Polerecky et al., 2012) in MATLAB. Symbiosis-specific C and N_2_ fixation rates were calculated for each association as in Montoya et al. (1996) (see Supplementary Material). The resulting cell-specific rates were compared between conditions with a MannWhitney test ( = 0.05).

## Results

### Experimental Conditions

We investigated the role of light as a direct and indirect (through the photosynthetic activity of the unicellular eukaryotic host) regulator of cellular processes in the diazotrophic cyanobacterial symbiont UCYN-A. Natural microbial populations from coastal surface water containing the UCYN-A2 symbiosis were incubated at *in situ* temperature and nutrient conditions under several dark/light scenarios using clear and opaque incubators (Figure 1, see also section Materials and Methods and Supplementary Figures 13). In control conditions, bulk C fixation (BCF) rates were 8 0.4 and 18 mmol C m^3^ d^1^ during days 1 and 2, respectively (Supplementary Figure 4). When incubations were kept under dark conditions, BCF rates were below the detection limit, indicating broad-scale inhibition of oxygenic photosynthesis at the community level. Switching incubation conditions back to normal day-night cycles after 24 h in the dark (DTL, Figure 1 and Supplementary Figure 1) resulted in recovery of BCF (28 5 mmol C m^3^ d^1^). In both DCMU conditions, BCF rates were greater than those in the dark and above detection limits, but the rates were negligible compared to control values (Supplementary Figure 4) despite identical light/dark conditions, indicating successful inhibition of oxygenic photosynthesis at the community level. In all conditions, bulk N_2_ fixation rates were below detection limits, which varied from 0.01 to 0.05 mmol N m^3^ d^1^ (Supplementary Table 1).

### UCYN-A2 Abundances and Replication

**FIGURE 2:**
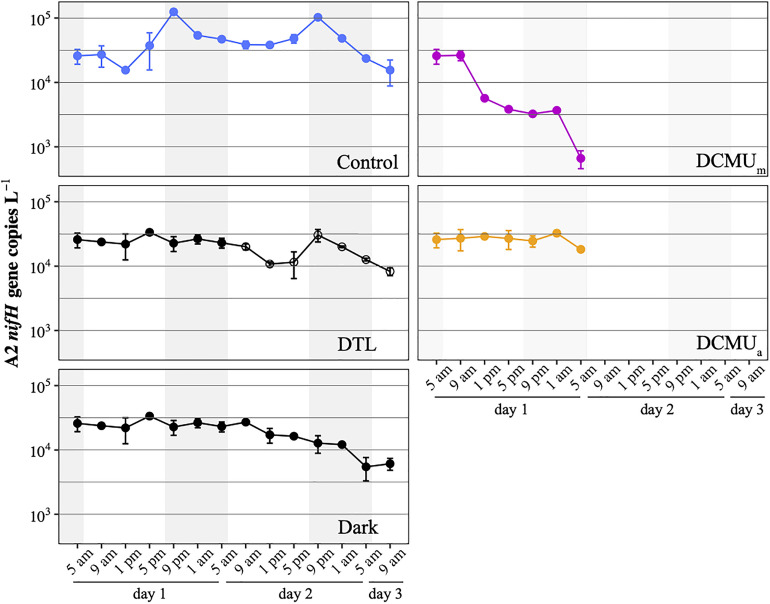
UCYN-A2 *nifH* gene abundances in control (blue), DTL (black and open circles), Dark (black), DCMU_*m*_ (purple), and DCMU_*a*_ (orange) incubations. Mean SD from duplicate incubations are shown at each time point. DCMU was spiked at 5 a.m. for the DCMU_*m*_ condition and at 1 p.m. for the DCMU_*a*_ condition. Note that the DTL condition data start at 9 a.m. on the second day of the experiment, and values plotted before that are identical to those from the Dark condition.

The natural microbial populations present in the waters at the SIO pier in May 2018 contained both UCYN-A1 and UCYN-A2 sublineages (Mills et al., 2020). Preliminary qPCR analyses showed that abundances were 10-fold higher for UCYN-A2 than for UCYN-A1, which constrained our analyses to the UCYN-A2 lineage. UCYN-A2 was present at 2 10^4^ *nifH* gene copies L^1^ at the onset of the experiment and remained at this magnitude during the 52-h long experiment in all conditions except when DCMU was added before sunrise (DCMU_*m*_, Figure 2). In DCMU_*m*_ incubations, the *nifH*-based abundance of UCYN-A2 was maintained for 4 h, then decreased throughout the remaining sampling points. By contrast, when DCMU was added at solar noon (1 p.m.; DCMU_*a*_), after the communities had experienced a full morning under control conditions, UCYN-A2 *nifH*-based abundances stayed close to initial values for the remaining daylight and the ensuing dark period (Figure 2).

**FIGURE 3:**
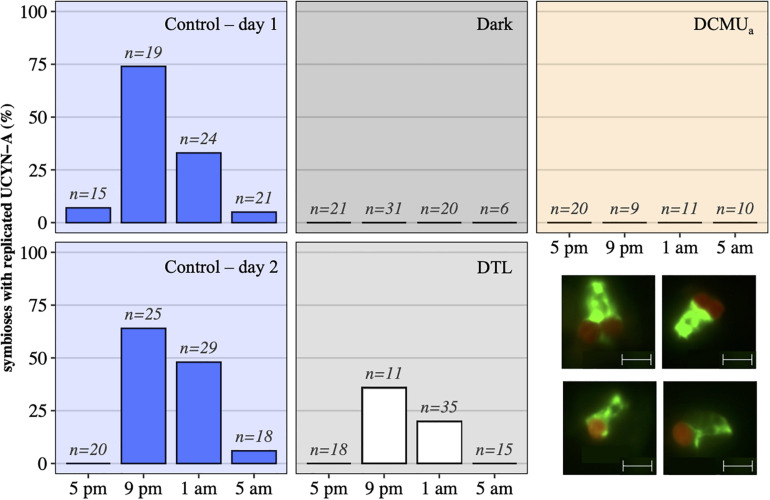
UCYN-A2 replication and division patterns in control (blue day 1, top and day 2, bottom), dark (black), DCMU_*a*_ (orange), and DTL (white) experimental conditions, as determined by epifluorescence microscopy. For each time point and condition, *n* represents the number of individual symbioses analyzed. Bottom right: examples of epifluorescence microscopy images showing individual symbioses where the host (green) is associated with a replicated symbiont (red) as seen in samples collected at 9 p.m. in the control (top row), or symbioses with a unique symbiont as seen in samples collected at 9 p.m. in the dark (bottom row). The scale indicates 5 m.

In control incubations, peak abundance was observed in the early evening at 9 p.m. (Figure 2). CARD-FISH visualization showed a high proportion (up to 74%) of host cells containing replicated UCYN-A2 cells at 9 p.m. compared to 5 p.m. (Figure 3), suggesting that the 9 p.m. increase in *nifH* gene copies L^1^ reflects symbiont DNA replication before cell division, which can be observed with microscopy. The number of host cells containing replicated UCYN-A2 cells declined in the early morning as the hosts presumably divided, returning to a UCYN-A2 to host cell ratio of 1:1 (Figure 3). The pattern was repeated over the two-day experiment, showing that the growth of the UCYN-A2 symbiosis was maintained in control conditions. After replication and division, UCYN-A2 abundances declined in the morning (Figure 2), possibly reflecting grazing losses as previously observed in other cyanobacterial and phytoplankton species such as *Prochlorococcus* (Ribalet et al., 2015). Contrasting with control conditions, UCYN-A2 replication was absent from all other experimental conditions except DTL (Figures 2, 3). These results indicate that the symbioses did not go through nighttime cell division in the absence of daytime light or when host photosynthesis was inhibited, regardless of the timing of DCMU addition. In the DTL treatment, both the peak in *nifH* gene copies L^1^ and the higher proportion of symbioses with replicated UCYN-A2 were observed at 9 p.m., although at a lower magnitude than in control incubations. These observations show that the return of a normal light period recovered UCYN-A2 DNA replication and cell division.

### UCYN-A2 *nifH* Transcriptional Patterns

**FIGURE 4:**
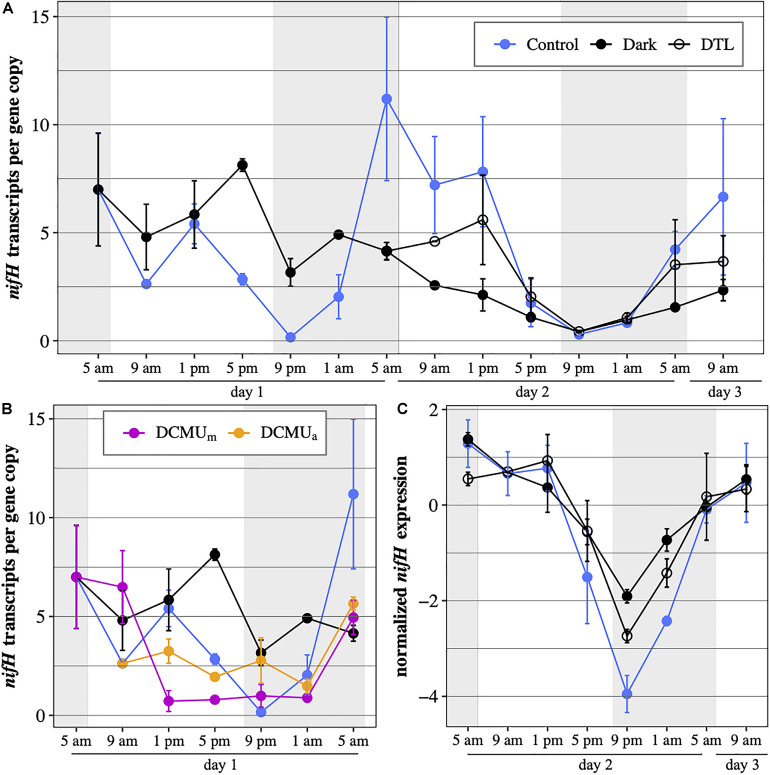
UCYN-A2 *nifH* gene expression in transcripts per gene copy, **(A)** during the full 52-h experiment in control incubations (blue), incubations returning to normal light-dark conditions after spending the first 24 h (DTL, open circles) or the first 48 h (Dark, black) of the experiment in the dark and **(B)** during the first 24 h of the experiment in control incubations (blue), dark incubations (black) and incubations exposed to DCMU added at 5 a.m. (purple) or at 1 p.m. (orange). **(C)** normalized UCYN-A2 *nifH* gene expression during the last 28 h of the experiment in control (blue), dark (black), and DTL (open circles) incubations. Expression levels were normalized to the median expression over that period within each condition and log_2_-transformed. Mean SD of biological duplicates are shown. Shaded areas represent nighttime.

In control conditions, UCYN-A2 *nifH* transcripts per gene copy generally reached maximum values from early morning to afternoon (5 a.m., 9 a.m., and 1 p.m.), and were minimal in the evening (5 p.m., 9 p.m., and 1 a.m.; Figure 4A) on both days. *nifH* continued to be transcribed under dark conditions but diverged from control patterns. While *nifH* transcripts per gene copy in control incubations dropped by half between 1 and 5 p.m., in the dark they remained close to expression levels detected in the morning. This resulted in expression levels three times higher in the dark compared to control incubations at 5 p.m. on the first day. *nifH* transcripts per gene copy in the dark were generally higher during the first 24 h, reflecting different responses to the absence of light after a normal light-dark cycle (day 1) or after 24 h of prolonged darkness (day 2). In the DTL incubations, *nifH* transcripts per gene copy increased from 5 a.m. to 1 p.m. compared to dark incubations and reached levels similar to those of control incubations (Figure 4A). While *nifH* transcript abundances varied among the different light-dark experimental conditions, the general diel pattern of *nifH* expression, i.e., the timing and trajectory of the oscillations, was conserved in all three experimental conditions, which was particularly evident on day 2 (RAIN, *p* 0.003, Figure 4C). Minimum expression was observed right after sunset (9 p.m.) on both days in all three experimental conditions.

In general, DCMU additions resulted in the lowest levels of *nifH* transcripts per gene copy (Figure 4B). There were minor, but possibly important, differences in the response to DCMU based on the timing of the addition. In DCMU_*m*_ incubations, *nifH* transcripts per gene copy at 9 a.m. remained elevated compared to control levels and similar to those measured in dark incubations. After the 9 a.m. time point, DCMU_*m*_ *nifH* transcripts per gene copy decreased dramatically, coinciding with the decrease in UCYN-A2 *nifH* abundance (Figure 2). In contrast, in the DCMU_*a*_ incubations UCYN-A2 maintained *nifH* transcription below control values but above those detected in DCMU_*m*_. In both DCMU conditions, *nifH* transcript levels remained relatively constant for 12 h between 1 p.m. and 1 a.m. Interestingly, transcript abundances increased in the late dark period between 1 a.m. and 5 a.m. similarly to the control, although at a smaller magnitude (Figure 4B).

### UCYN-A2 Symbiosis Cell-Specific C and N_2_ Fixation Rates

**FIGURE 5:**
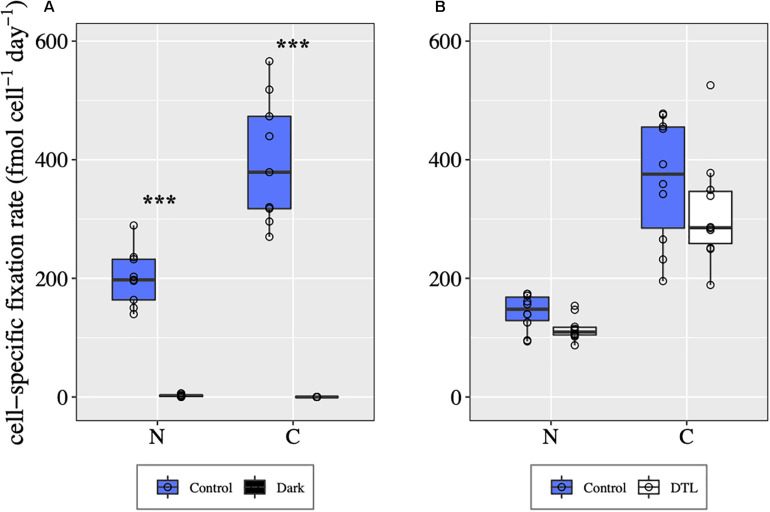
UCYN-A symbiosis cell-specific C and N_2_ fixation rates in different light-dark conditions during day 1 **(A)** and day 2 **(B)**, determined for individual UCYN-A2/haptophyte symbiotic assemblages. For each condition, the values shown for C fixation and for N_2_ fixation were obtained from the same n cells, with *n* = 10 for each condition except for the control from day 1 for which *n* = 9. ***indicates significant differences between conditions (*p* < 0.0001) as determined by a MannWhitney test.

In control conditions, all UCYN-A2 symbioses analyzed were fixing C and N_2_ during both days 1 and 2 (Figure 5). In the dark, host C fixation was not detected, and UCYN-A2 N_2_ fixation was negligible compared to control values (MannWhitney test, *p* < 0.00001, Figure 5A). In the DTL treatment, UCYN-A2 symbiosis cell-specific C and N_2_ fixation rates were comparable to the control rates (Figure 5B), indicating recovery of these processes once the light period resumed. Sufficient numbers of cells could not be found from the DCMU_*m*_ treatment for CARD-FISH analysis and cell-specific rate measurement.

## Discussion

This study was designed to investigate mechanisms involved in the daily cycles of N_2_ fixation, C fixation and cell division in the UCYN-A haptophyte symbiosis, by manipulating the light-dark cycle and interrupting host oxygenic photosynthesis. The experimental setup mimicked natural conditions and results from the control incubations showed steady UCYN-A2 abundances, maximum UCYN-A2 *nifH* expression and N_2_ fixation during the day, and symbiont division early during the night, consistent with previous *in situ* observations on this lineage and others (Church et al., 2005b; Turk et al., 2011; Krupke et al., 2013; Muoz-Marn et al., 2019). Through alteration of dark-light cycles, we show that light is required to observe typical activity patterns in the symbiont, from molecular processes such as *nifH* expression patterns, to functional traits such as active diazotrophy and cell replication and division. Using a PSII inhibitor targeting the eukaryotic host we also show that the haptophyte partner, through its photosynthetic activity, could be an important mediator of the symbiont dependence on light for these processes.

### Coupling of Light, Host Carbon Fixation and Symbiont N_2_ Fixation

Our results demonstrate that light is necessary, as an energy source or as a signal, for N_2_ fixation in UCYN-A2. The timing of N_2_ fixation could not be directly assessed since our incubations were run for 24 h. However, (1) under constant dark conditions there was no N_2_ fixation, and (2) N_2_ fixation returned to control levels once cells were exposed to light after extended dark incubations. Together these results show a strong temporal connection between N_2_ fixation in UCYN-A2 and the presence of a light period.

There are several potential explanations for the coupling between light and UCYN-A N_2_ fixation evidenced in our study. First, light may play a direct role in UCYN-A physiology. Daytime light-dependent N_2_ fixation by UCYN-A is an unusual feature since closely related, free-living unicellular N_2_-fixing species only fix during the dark period to avoid photosynthetic oxygen. Although UCYN-A has lost PSII and the Calvin cycle, it has retained the PSI genes which are transcribed in a diel cycle similar to that of *nif* genes (Muoz-Marn et al., 2019). The persistence of PSI in this organism appears to be important since similar freshwater symbiotic unicellular diazotrophic cyanobacteria have lost both photosystems (Nakayama et al., 2014; Nakayama and Inagaki, 2017). PSI may play an important role in UCYN-A metabolism to generate energy or reductant to fuel N_2_ fixation and may provide protection from oxygen using the Mehler reaction (Milligan et al., 2007). Thus, the activity of PSI could be essential to N_2_ fixation in UCYN-A and explain the atypical timing of this process in this unicellular cyanobacterium.

Our results could also be interpreted as evidence that UCYN-A N_2_ fixation is dependent on freshly fixed photosynthate or signals from the host. Both sequenced UCYN-A genomes lack the genes necessary for carbon fixation (Tripp et al., 2010; Bombar et al., 2014), implicating a dependence on organic carbon exogenous to the symbiont. Transfer of fixed carbon from the host to the symbiont has been demonstrated in the UCYN-A1 and UCYN-A2 lineages (Thompson et al., 2012; Martnez-Prez et al., 2016). The timing of transfer of host-derived organic carbon, the chemical form that is transferred, and its contribution to the energy pool supporting N_2_ fixation by UCYN-A have not yet been resolved. Our study shows parallel responses of C and N_2_ fixation to continued incubation in the dark, during which both processes are shut down, and to the reintroduction of a light period, where both processes resume to levels comparable to control values. In a study of the freshwater diatom *Rhopalodia gibba*, light was also found to be indispensable for N_2_ fixation by the diatoms cyanobacterial endosymbiont (Prechtl et al., 2004). This organism, which is closely related to UCYN-A (Mare et al., 2019), does not have the genes required to assemble either photosystem or to perform carbon fixation (Nakayama and Inagaki, 2017). In that case, it is very likely that the symbionts requirement for light illustrates a time-sensitive reliance on host photosynthate as a source of energy to fuel daytime N_2_ fixation. Our study suggests that a similar scenario could be at play for UCYN-A and might be a fundamental characteristic of symbioses involving N_2_-fixing unicellular cyanobacteria and photosynthetic hosts.

Finally, daytime N_2_ fixation in UCYN-A could be an adaptation that increases the fitness of the haptophyte host rather than that of the symbiont. It is possible that daytime N_2_ fixation by UCYN-A provides the host with an ample supply of fixed N necessary to sustain its own daytime metabolic needs, and to maximize C fixation (Krupke et al., 2015). This fits the more general hypothesis that the atypical features of UCYN-A are driven by a tight metabolic embedding with the haptophyte host, which implies that the host at least partially controls some cellular processes in the symbiont. The tight coupling between N_2_ and C fixation with the presence of light requires mechanisms to regulate the timing and implementation of these processes in UCYN-A and its host.

### Transcriptional Controls in UCYN-A: Possible Roles of Light and of the Eukaryotic Host

Results from our study also show that transcriptional activity in UCYN-A is modulated by mechanisms involving light, either directly or through host activity. While both C and N_2_ fixation were interrupted in the dark treatment, *nifH* gene transcription continued. However, differences in transcript levels compared to control conditions indicate that the presence of light is also required to observe regular transcriptional patterns in the symbiont. In the dark, *nifH* transcripts levels measured at dawn persisted throughout daytime, showing that light, either as a direct trigger or through the metabolic activities it enables, is necessary for the decrease observed after the morning peak in control conditions. Dampened transcript levels in the dark on day 2 and the rapid return of control-like levels of *nifH* gene transcripts in the light after the extended dark period (DTL) also suggest that *nifH* transcription in UCYN-A is influenced by energy and reductant availability.

UCYN-A2 *nifH* expression also deviated from control patterns when host photosynthesis was chemically inhibited (DCMU_*m*_ and DCMU_*a*_) while a regular light-dark cycle was maintained for those incubations. Because DCMU is a specific inhibitor of PSII, which UCYN-A lacks, the symbiont response to the presence of DCMU can be attributed to changes in host physiology. In our study, the similarities in the short-term (5 a.m. 9 a.m.) transcriptional response to darkness and blocked host photosynthesis suggest that in the morning, light-dependent host activity rather than light itself could be an important regulator of the daytime transcription patterns normally observed in the symbiont for *nifH*, and conceivably other genes. A recent model-based study predicts that a minimum of 28 metabolites synthesized by the host are necessary for growth and activity of the symbiont (Sarkar et al., 2021). The amount provided as well as the timing of delivery of these compounds by the host may act as triggers of transcriptional regulations in the symbiont. Our observation that in the dark UCYN-A2 maintained pre-dawn *nifH* expression levels until nighttime is consistent with the hypothesis that freshly synthesized and transferred host metabolites are involved in a regulation cascade driving *nifH* transcript levels down later in the day. Our results provide evidence that UCYN-A activity is controlled in part via host metabolism. However, during the second portion of the daytime, *nifH* transcriptional patterns differed between dark and in DCMU incubations, suggesting that blocked host photosynthesis doesnt explain on its own the divergence from control observed in dark incubations. Thus, while the host appears to influence gene expression in the symbiont, other mechanisms may also be at play.

### Insight Into Circadian Rhythms in the Symbiosis

Metabolic activities in cyanobacteria are under the control of a circadian clock that enhances fitness by enabling cells to anticipate daily changes in environmental conditions. In UCYN-A, clear diel oscillations in transcript abundance have been observed for most genes (Muoz-Marn et al., 2019), a surprising result considering that 2 of the 3 *kai* genes responsible for the circadian oscillator in cyanobacteria (Ishiura et al., 1998) are missing from the UCYN-A genomes (Tripp et al., 2010; Bombar et al., 2014). In our study, diel oscillations in *nifH* expression were observed in all experimental conditions, including in dark incubations where *nifH* expression was lower compared to control conditions on day 2. The typical increase in *nifH* transcript abundance before sunrise was observed in all incubations, including in DCMU incubations where inhibited host photosynthesis was associated with unusually steady *nifH* transcript levels during most of the daytime. These observations show a link between the patterns of *nifH* expression in UCYN-A2 and the time of day regardless of light conditions and host status, suggesting that a circadian clock may still be controlling gene transcription in the symbiont.

It is unclear what mechanisms allow for circadian rhythms in UCYN-A in the absence of the oscillator proteins KaiAB. In the marine cyanobacterium *Prochlorococcus*, which does not possess the *kaiA* gene (Garczarek et al., 2001; Rocap et al., 2003), the remaining KaiBC proteins maintain their function through biochemical adaptations that compensate the loss of the KaiA protein (Axmann et al., 2009), leading to a conserved, though less robust, circadian clock (Holtzendorff et al., 2008). Similarly, it is possible that in UCYN-A, the remaining clock components are still operational in the absence of *kaiAB* through unique adaptations that could be related to the symbiotic lifestyle of UCYN-A. Eukaryotic algae have long been known to also have circadian clocks themselves (Suzuki and Johnson, 2001), and daily cycles in UCYN-A could be under host control. Interestingly, diel variations in energetic status are the primary mechanism through which the cyanobacterial kai oscillator is entrained (Rust et al., 2011; Pattanayak et al., 2014). For example, pulses of glucose are sufficient to entrain the clock in the absence of light cues in the model cyanobacterial strain *Synechococcus elongatus* (Pattanayak et al., 2015). Thus, if the remaining clock components in UCYN-A are functional, their activity is intrinsically linked to the host as the main provider of fixed carbon to the symbiont. It is probable that multiple parameters, related to the presence of light and variations in host activity and resulting energy levels throughout the day, together contribute to the clock-like orchestration of the daily patterns of transcription and activity in UCYN-A. Future experiments including a constant light treatment are required for definitively evaluating the question of circadian control of gene expression in UCYN-A, and will be facilitated when cultures of the UCYN-A symbiosis become available. Results from our study provide valuable insight and will help form future experiments regarding the existence of such a clock and mechanisms involved.

### Evidence of Host Control on UCYN-A Growth and Cell Division

Our experiment provides the strongest evidence to date that replication and division in UCYN-A are at least partially under host control. As in previous studies (Cabello et al., 2016; Muoz-Marn et al., 2019), we observed that in control conditions UCYN-A2 cells divided in early evening, going from a single symbiont to two prior to host division later in the night. Incubation in the dark led to no observable replication or division of the symbiont, showing that the cell cycle is disrupted without light. Return to light following extended dark restored division at the expected timing, although the number of cells proceeding to division decreased. The importance of dark-light cycles for cell replication is documented in other microorganisms, such as *Prochlorococcus*, which replicates in the afternoon and divides at night (Vaulot et al., 1995). In *Prochlorococcus*, the absence of a light period results in cells arrested in the G1 phase of the cell cycle, before DNA is replicated (Jacquet et al., 2001). In our experiment, the division of UCYN-A2 was also disrupted when host photosynthesis was chemically inhibited in regular dark-light cycles conditions. It is particularly striking that symbiont division was disrupted when DCMU was added midday after a full morning in control conditions. These observations suggest that while light may be involved in cell cycle regulation, it alone is not sufficient for UCYN-A division to proceed; division of the symbiont requires a photosynthetically active host. If the symbiosis between UCYN-A and the haptophyte is obligatory, tight control mechanisms must exist to ensure synchronized growth between the two partners.

## Conclusion

Our results provide a detailed picture of how light and the photosynthetic activity of the haptophyte host affect daily processes such as gene transcription, N_2_ fixation and cell division in the symbiotic cyanobacterium UCYN-A2. Additional experiments in more controlled settings, particularly once cultures of the symbiosis become available, are needed to elucidate the specific mechanisms through which light and the physiology of the haptophyte host control UCYN-A activity. The hypotheses generated by this study about the functioning of the UCYN-A-haptophyte symbiosis are based on results obtained from the UCYN-A2 lineage. At least five ecologically distinct UCYN-A sublineages have been identified to date, with specific global distribution patterns (Turk-Kubo et al., 2017). While functional differences might be identified in the future, existing data indicate strong similarities across the sublineages and a conserved function organized around the exchange of fixed N and C between the cyanobacterial and haptophyte partners (Bombar et al., 2014; Cornejo-Castillo et al., 2016). The existence of core functional traits within the UCYN-A lineage suggests that some, if not most, regulatory mechanisms at play in the UCYN-A2 sublineage are shared by other members of the group. Future work will clarify whether the patterns observed in our study with the UCYN-A2 lineage also hold for other UCYN-A lineages. Our study provides a framework for a better understanding of the unique features that shape this abundant, ecologically important N_2_-fixing symbiotic association.

## Data Availability Statement

The raw data supporting the conclusions of this article will be made available by the authors, without undue reservation.

## Author Contributions

ML and JZ designed the study. ML, KT-K, FC-C, and BH ran the experiments and performed sampling. ML and KT-K performed sample and data analyses. ML, KT-K, and JZ wrote the manuscript with edits from BH and FC-C. All authors contributed to the article and approved the submitted version.

## Conflict of Interest

The authors declare that the research was conducted in the absence of any commercial or financial relationships that could be construed as a potential conflict of interest.
