# Supplementary material for: Critical Role of Light in the Growth and Activity of the Marine N2-Fixing UCYN-A Symbiosis
Source: Front Microbiol. 2021 May 5;12:666739. doi: 10.3389/fmicb.2021.666739 (PMC8139342; doi:10.3389/fmicb.2021.666739)
Supplement: Supplementary file 2 [file Data_Sheet_1.PDF]

Supplementary Methods

Preparation of $^{15}N_2$-enriched seawater – Seawater collected from the experimental site was sterile filtered through a Pall 0.2 µm Acropak 1550 capsule filter (Pall Corp, Port Washington, NY, USA). The filtered seawater was degassed under vacuum for >1 hr while being stirred. Degassed water was transferred via siphon into 2L PC bottles and capped with PTFE-lined septa and caps (Ace Glass Incorporated, Vineland, NJ). Bottles were then over pressurized by injecting ca. 20 cc of $^{15}N_2$ gas (Cambridge Isotope Laboratories, Tewksbury, MA, USA) and agitated at room temperature on a rocking plate for >12 hrs.

Double CARD-FISH assay – Filters were embedded in 0.1% agarose and incubated with lysozyme (400 U µL^{-1} final concentration) at 37°C for 1 hour. Filter sections were cut and incubated at 46°C overnight with the UBRADO69 probe, targeting the UCYN-A2 host *Braarudosphaera bigelowii,* and helper probes A-PRYM and B-PRYM (Cabello et al., 2016; Cornejo-Castillo et al., 2016) each at 5 ng µL^{-1} in hybridization buffer. Competitors were not used, as tests showed a clear size difference between the UCYN-A1 and UCYN-A2 sublineages present in these samples, allowing to visually differentiate between them. After the first hybridization, filter sections were washed (56mM NaCl, 5mM EDTA, 0.01% SDS, 20mM Tris-HCl pH 8) for 20 minutes at 48°C and equilibrated in 1X PBS for 15 minutes at room temperature. Amplification of the signal was achieved by incubating the filter sections with Alexa 488-labeled tyramide (4 µg mL^{-1}) in amplification buffer in the dark at 46°C for 30 minutes. Filter sections were rinsed with PBS and transferred to 0.01 M HCl for 10 minutes in the dark to inactive probe peroxidases. For hybridization of the symbiont, filter sections were incubated for 3 hours at 35°C using the UCYN-A2 732 probe (Cornejo-Castillo et al., 2016) along with helper probes Helper A-732 and Helper B-732 (Krupke et al., 2013) each at 0.16 ng µL^{-1} in hybridization buffer. Sections were washed at 37°C for 20 minutes using the same washing buffer as described above (except for NaCl, 9 mM final concentration). Signal amplification was performed in the dark at 46°C for 30 minutes using CY-3-labeled tyramide in amplification buffer. Filter sections were washed in 1X PBS and water, air dried then mounted on microscopy glass slides using an antifading solution mixed with 4′,6-diamidino-2-phenylindole (DAPI, 5 µg mL^{-1}).

Symbiosis-specific C and N_2 fixation rates calculations – For each individual UCYN-A2-host association, the 30 planes were aligned, accumulated and regions of interest (ROIs) were defined for each association. Total cell volume (including host and symbiont) was derived from the diameter measured for each symbiosis, assuming a spherical shape. Two ratios were obtained per symbiosis: ^{13}C enrichment in atom% ($^{13}C/(^{13}C+^{12}C)$) and ^{15}N enrichment in atom% ($^{12}C^{15}N/(^{12}C^{14}N+^{12}C^{15}N)$). Symbiosis-specific C and N_2 fixation rates were calculated for each association using the formulas:

$$NFR_{cell} = \left(\frac{(a\%15_N)_{T24} - (a\%15_N)_{T0}}{\left(a\%15_{N_2}\right)_{T0} - (a\%15_N)_{T0}} \right) \times PN$$

And

$$CFR_{cell} = \left(\frac{(a\%13_C)_{T24} - (a\%13_C)_{T0}}{\left(a\%13_{HCO_3-}\right)_{T0} - (a\%13_C)_{T0}} \right) \times PC$$

Where:

NFR_{cell} is the N_2 fixation rate per symbiosis, in fmol N $cell^{-1}$ day^{-1},

$\boldsymbol{CFR_{cell}}$ is the C fixation rate per symbiosis, in fmol C $cell^{-1}$ day^{-1},

$\boldsymbol{(a\%15_N)_{T24}}$ is the mean ^{15}N enrichment (in atom%) of a given host-symbiont association after a 24-hour incubation in $^{15}N_2$/ $NaH^{13}CO_3$-enriched seawater, as determined by nanoSIMS,

$\boldsymbol{(a\%13_C)_{T24}}$ is the mean ^{13}C enrichment (in atom%) of a given host-symbiont association after a 24-hour incubation in $^{15}N_2$/ $NaH^{13}CO_3$-enriched seawater, as determined by nanoSIMS,

$\boldsymbol{(a\%15_N)_{T0}}$ is the mean ^{15}N enrichment (in atom%) of host-symbiont associations (n=5) collected at T_0 prior to $^{15}N_2/NaH^{13}CO_3$ enrichment, as determined by nanoSIMS,

$\boldsymbol{(a\%13_C)_{T0}}$ is the mean ^{13}C enrichment (in atom%) of a given host-symbiont association after a 24-hour incubation without enrichment, as determined by nanoSIMS,

$\boldsymbol{\left(a\%15_{N_2}\right)_{T0}}$ is the average $^{15}N_2$ enrichment (in atom%) of seawater at the beginning of the incubations, calculated based on the $^{15}N_2$ enrichment measured by MIMS analysis in the $^{15}N_2$/ $NaH^{13}CO_3$-enriched seawater used to spike the incubations and accounting for volume dilution,

$\boldsymbol{\left(a\%13_{HCO_3-}\right)_{T0}}$ is the average $H^{13}CO_3$ enrichment (in atom%) of seawater at the beginning of the incubations after the spiking of $^{15}N_2$ and $NaH^{13}CO_3$-enriched seawaters, where

ambient dissolved inorganic carbon concentration was calculated to be 2.12 mM using ambient salinity (Weiss, 1974),

PN and PC are the estimated N and C content of the host-symbiont association, respectively, determined based on cell volume calculated from nanoSIMS image analysis, from which cell C and N content were derived using a previously published equation (Strathmann, 1967; Krupke et al., 2013) describing the relationship between C content and biovolume in haptophytes, and a C:N ratio of 6.3 for the symbiosis (Martínez-Pérez et al., 2016).

References

Cabello, A. M., Cornejo-Castillo, F. M., Raho, N., Blasco, D., Vidal, M., Audic, S., et al. (2016). Global distribution and vertical patterns of a prymnesiophyte–cyanobacteria obligate symbiosis. *ISME J.* 10, 693–706. doi:10.1038/ismej.2015.147.

Cornejo-Castillo, F. M., Cabello, A. M., Salazar, G., Sánchez-Baracaldo, P., Lima-Mendez, G., Hingamp, P., et al. (2016). Cyanobacterial symbionts diverged in the late Cretaceous towards lineage-specific nitrogen fixation factories in single-celled phytoplankton. *Nat. Commun.* 7, 11071. doi:10.1038/ncomms11071.

Krupke, A., Musat, N., LaRoche, J., Mohr, W., Fuchs, B. M., Amann, R. I., et al. (2013). In situ identification and N_2 and C fixation rates of uncultivated cyanobacteria populations. *Syst. Appl. Microbiol.* 36, 259–271. doi:10.1016/j.syapm.2013.02.002.

Martínez-Pérez, C., Mohr, W., Löscher, C. R., Dekaezemacker, J., Littmann, S., Yilmaz, P., et al. (2016). The small unicellular diazotrophic symbiont, UCYN-A, is a key player in the marine nitrogen cycle. *Nat. Microbiol.* 1, 1–7. doi:10.1038/nmicrobiol.2016.163.

Strathmann, R. (1967). Estimating the organic carbon content of phytoplankton from cell volume or plasma volume. *Limnol. Oceanogr.* 12, 411–418. doi:10.4319/lo.1967.12.3.0411.

Weiss, R. F. (1974). Carbon dioxide in water and seawater: the solubility of a non-ideal gas. *Mar. Chem.* 2, 203–215. doi:10.1016/0304-4203(74)90015-2.

Supplementary Figure Legends

Supplementary Figure 1 – Schematic experimental design. Duplicate bottles are shown with colors corresponding to treatments, and position in the figure corresponding to the time of sampling as indicated on the bottom arrow showing the experimental timeline. Blue corresponds to control bottles (incubated in regular light/dark conditions), purple corresponds to $DCMU_m$ bottles (spiked with 100 µg L^{-1} DCMU at 5 a.m. on day 1 and incubated in normal light/dark conditions for 24 hours), orange corresponds to $DCMU_a$ bottles (incubated in normal light/dark conditions and spiked with 100 µg L^{-1} DCMU at 1 p.m. on day 1), and gray corresponds to dark bottles (incubated in opaque containers). Colored, darker bottles with arrows represent bottles spiked with $^{15}N_2/H^{13}C0_3$- enriched seawater. The beginning of the arrow shows the time of enrichment (5 a.m. either on day 1 or on day 2), and the position of the bottle indicates the time of sampling. Dotted bottles represent bottles first incubated in opaque containers then transferred (at 5 a.m. on day 1, DTL, or on day 2, Dark) to clear containers where they were further incubated then sampled. The position of the dotted bottles represents sampling time. This experimental design was implemented for 2 L bottles (for RNA/DNA sampling and bulk N_2 and C fixation rates, see Methods) as well as 1 L bottles (for CARD-FISH and nanoSIMS sampling, see Methods).

Supplementary Figure 2 – temperature in the experimental incubators. Boxplots showing daytime (left) and nighttime (right) temperatures measured in the incubators (clear, 1 to 5; and opaque, 6 to 8) as compared to air temperature (A1 and A2). Mean is shown as navy diamonds.

Supplementary Figure 3 – daytime light levels in the experimental incubators. Light values collected from the 8 experimental incubators were compared to incident light measured at the surface of 2 of the 8 incubators, using data collected every minute for the 52 hours of the experiment. Boxplots show light levels per individual incubator during the daytime as a percent of incident light. The average light level in the clear incubators (1 to 5) was also calculated at one-minute intervals. Incubators 6 to 8 were opaque. Red diamonds represent the mean daytime value in each incubator and for the average of clear incubators.

Supplementary Figure 4 – Community carbon fixation rates. Inset graph shows values for Dark, DCMUm and DCMUa conditions on day 1 on a different scale. Dotted lines represent limit of detection. Mean ± SD of biological triplicates are shown when available. Note that the values for control and dark conditions on day 2 were obtained from a unique biological sample, as the POC content of the other replicates was too high for accurate ^{13}C isotope quantification.
