# Supplementary figures and images for: Critical Role of Light in the Growth and Activity of the Marine N2-Fixing UCYN-A Symbiosis
Source: Front Microbiol. 2021 May 5;12:666739. doi: 10.3389/fmicb.2021.666739 (PMC8139342; doi:10.3389/fmicb.2021.666739)

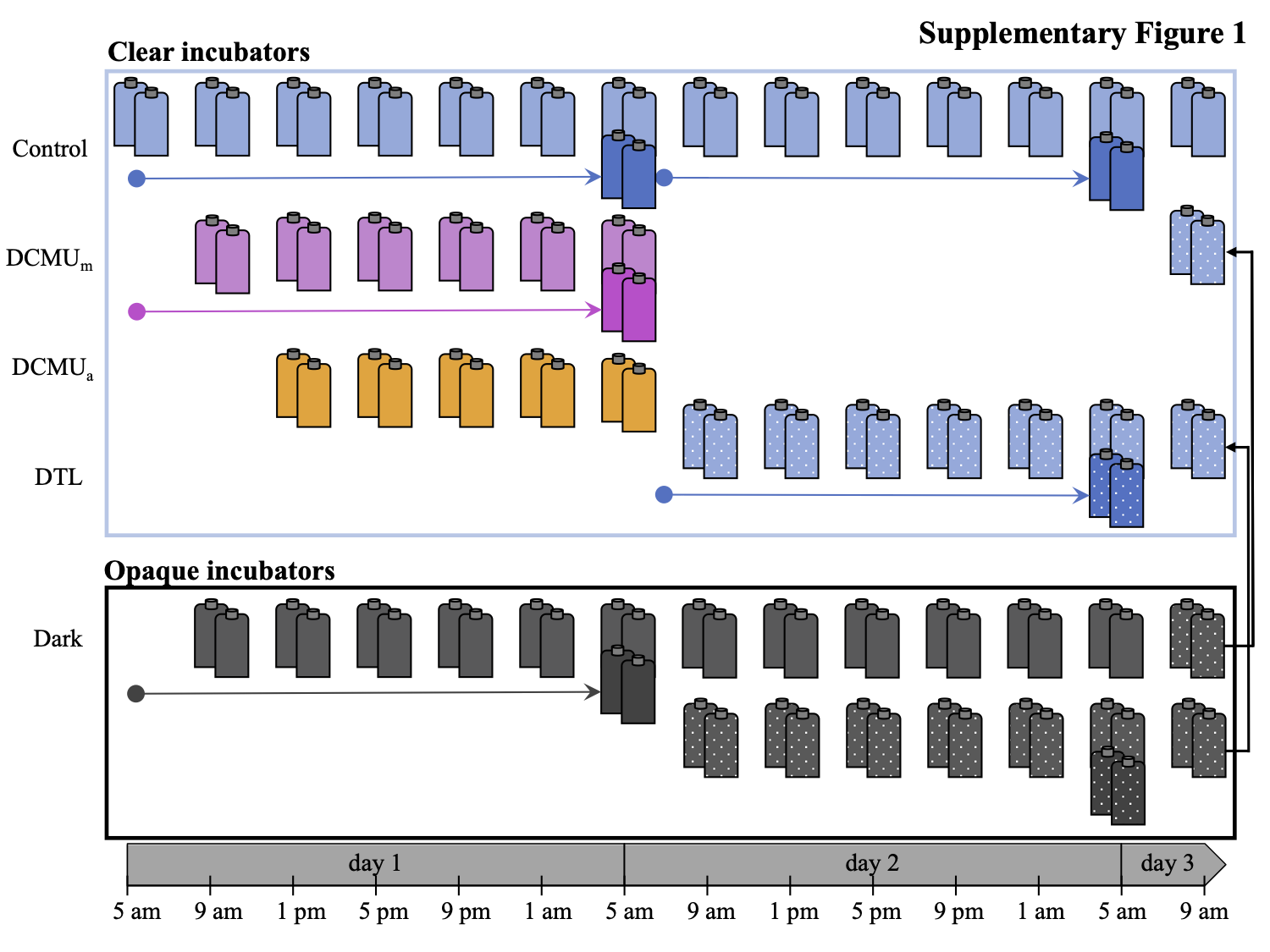

Supplement: Supplementary file 3 [file Image_1.TIFF]

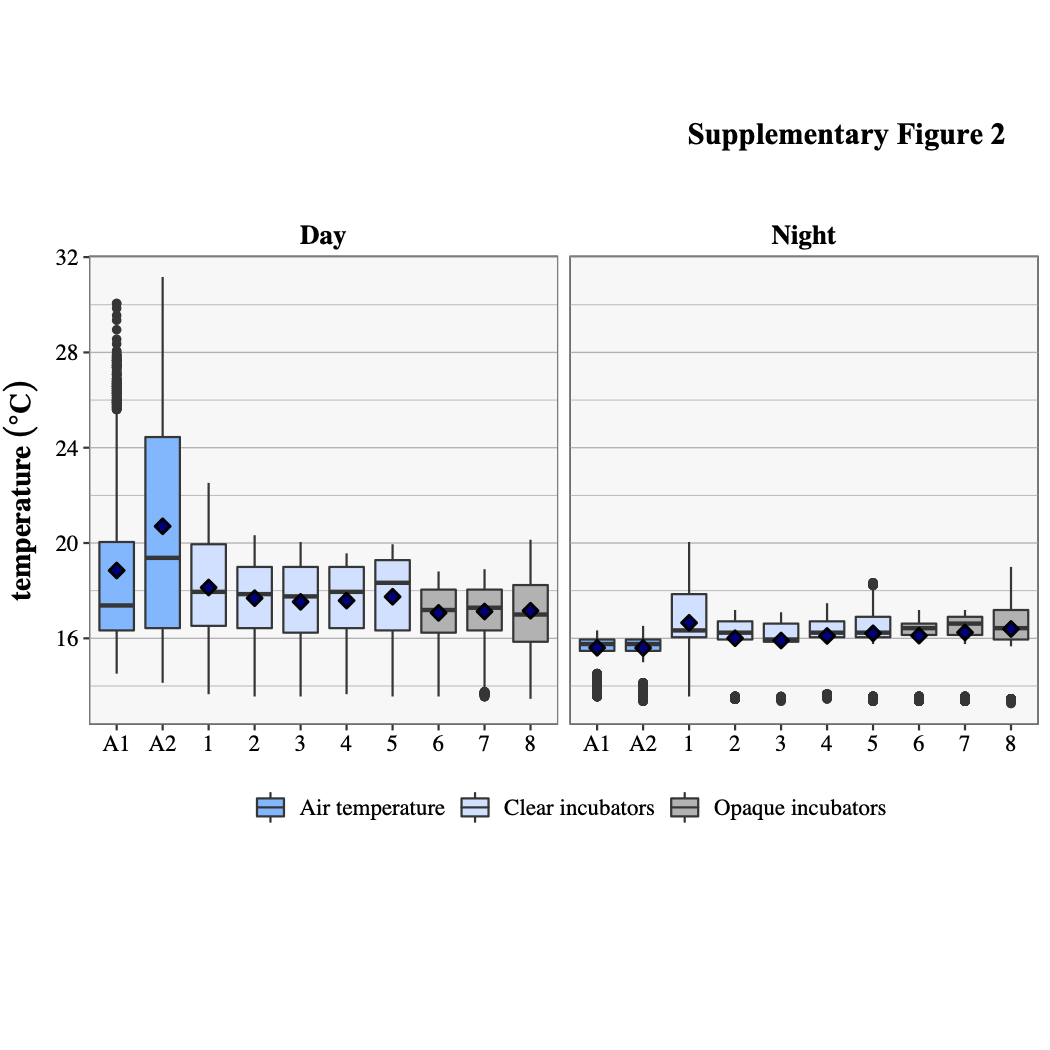

Supplement: Supplementary file 4 [file Image_2.TIFF]

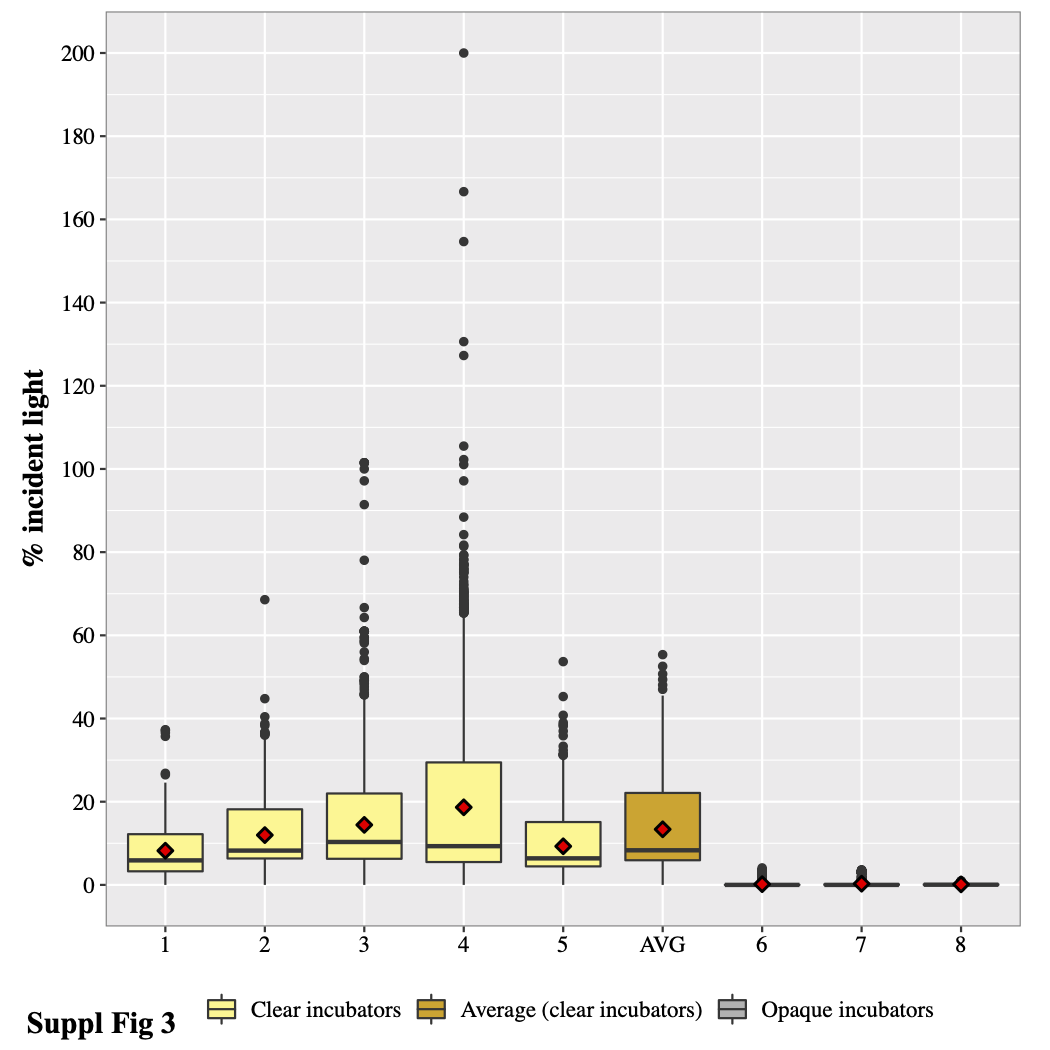

Supplement: Supplementary file 5 [file Image_3.TIFF]

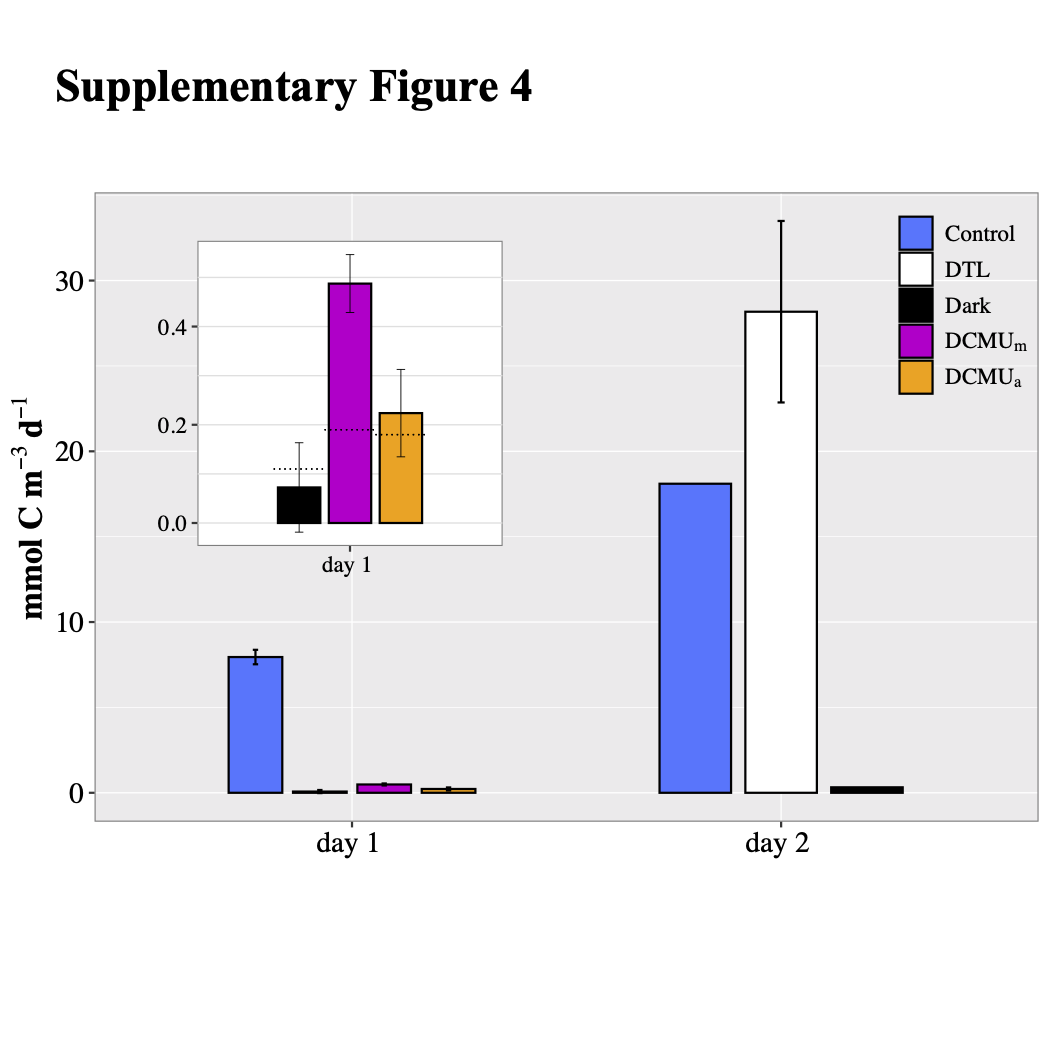

Supplement: Supplementary file 6 [file Image_4.TIFF]
